# Posterior reversible leukoencephalopathy syndrome (PRES) after kidney transplantation: a case report

**DOI:** 10.1590/1678-4685-JBN-3825

**Published:** 2018-04-19

**Authors:** Carla Beatriz Davi, Bruna Pinheiro de Moraes, Bruno Fontes Lichtenfels, João Batista Saldanha de Castro, Marcelle Maria Portal, Rosangela Munhoz Montenegro, Roberto Ceratti Manfro

**Affiliations:** 1 Universidade Federal do Rio Grande do Sul, Faculdade de Medicina, Hospital de Clínicas de Porto Alegre, Porto Alegre, RS, Brasil.

**Keywords:** Posterior Leukoencephalopathy Syndrome, Kidney Transplantation, Tacrolimus, Síndrome da Leucoencefalopatia Posterior Reversível, Transplante de Rim, Tacrolimus

## Abstract

**Introduction:**

Posterior reversible leukoencephalopathy syndrome (PRES) was first described by Hinchey in 1996. The syndrome is characterized by altered level of consciousness, headache, visual changes, and seizures associated with a vasogenic edema of the white matter that occurs predominantly in the occipital and parietal lobes. Imaging tests such as computed tomography (CT) and especially magnetic resonance imaging (MRI) support the diagnosis.

**Case Report:**

We report a case of a 48-year-old female patient who underwent a deceased donor kidney transplant and received tacrolimus as a part of the immunosuppressive regimen. Five weeks after transplantation she was admitted to the emergency due to sudden onset of confusion, disorientation, visual disturbances, and major headache. PRES was suspected and the diagnosis confirmed by brain MRI. Tacrolimus was withdrawn and rapid improvement of the neurological signs occurred leading to the conclusion that this drug triggered the syndrome.

**Conclusion:**

PRES is an unusual complication after organ transplantation and should be considered in the appropriate clinical setting. Physicians must be aware of this condition in order to provide early detection and appropriate treatment since delay in removing the cause may lead to permanent sequelae.

## RESUMO

**Introdução:**

A síndrome da leucoencefalopatia posterior reversível (PRES) foi descrita pela primeira vez por Hinchey, em 1996. A síndrome é caracterizada por nível alterado de consciência, cefaleia, alterações visuais e convulsões associadas a edema vasogênico da substância branca, oriundo predominantemente nos lobos occipital e parietal. Exames de imagem como tomografia computadorizada (TC) e em particular a ressonância magnética (MRI) corroboram o diagnóstico.

**Relato de caso:**

relatamos o caso de uma paciente do sexo feminino de 48 anos submetida a transplante renal com doador falecido em regime imunossupressor com tacrolimus. Cinco semanas após o transplante, a paciente deu entrada no pronto-socorro por conta de aparecimento súbito de confusão, desorientação, distúrbios visuais e cefaleia intensa. As suspeitas diagnósticas recaíram sobre PRES, e a confirmação foi feita por imagens de ressonância magnética do cérebro. Tacrolimus foi suspenso e os sinais neurológicos da paciente melhoraram rapidamente, indicando que o medicamento desencadeou a síndrome.

**Conclusão:**

PRES é uma complicação incomum após o transplante de órgãos e deve ser considerada em ambiente clínico adequado. O corpo clínico deve estar ciente dessa patologia, de modo a detectá- -la precocemente e dar início ao tratamento, uma vez que atrasos na eliminação da causa podem levar a sequelas permanentes.

## INTRODUCTION

Posterior reversible leukoencephalopathy syndrome (PRES) was first described by Hinchey in 1996.^1^ The syndrome is characterized by the occurrence of encephalopathy that may present with a variety of signs and symptoms including headache, altered vision, decreased visual acuity, cortical blindness, confusion, stupor, seizures, and hallucinations.^1^^,^ 2 It is associated with vasogenic edema of the white matter, predominantly in the occipital and parietal lobes,^5^^,^ 6 however, its pathophysiology is not yet fully understood. It is believed that endothelial dysfunction and alterations of cerebral autoregulation are involved.^1^ PRES is usually associated with hypertensive encephalopathy, eclampsia, and use of immunosuppressive drugs, particularly calcineurin inhibitors; the majority of the patients present with marked elevation of blood pressure.^1^

Here, we present a case of PRES in a kidney transplant recipient receiving tacrolimus in the immunosuppressive regimen, and emphasize the importance of early diagnosis and therapeutic measures.

## CASE REPORT

A 48-year-old white female with end-stage renal disease due to adult polycystic kidney disease was admitted for deceased donor kidney transplantation in April 2015. She had started renal replacement therapy with hemodialysis 7 years before and was in good general health with no significant co-morbidities. The donor was a 2-year-old female who suffered anoxic encephalopathy. At organ retrieval, donor serum creatinine was 0.58 mg/dL. Donor and recipient presented 4 HLA (ABDR) mismatches, cross matching was negative and no anti-donor specific HLA antibodies were found in the recipient’s serum. The kidneys were implanted “en bloc” and the transplant was performed after 18 hours of cold ischemia in static preservation on Euro-Collins solution. Immunosuppressive regimen consisted of Basiliximab® induction, tacrolimus, sodium mycophenolate, and steroids. The graft presented immediate function and the patient was discharged at post-operative day 36 (POD). During hospitalization, she presented a urinary tract infection and was submitted to antibiotic treatment for 10 days. The blood tacrolimus level three weeks before discharge was 15 µg/mL (receiving tacrolimus 7 mg twice daily orally). The dosage was immediately reduced to 5 mg twice daily, and three days after dose adjustment the blood level was 11.4 µg/mL; a new dose adjustment to 4 mg twice daily was done. At discharge, serum creatinine was stable at 1.6 mg/dL and blood tacrolimus level around 10 µg/mL. On the 19th POD, sodium mycophenolate was replaced by azathioprine due to severe diarrhea not responsive to dose fractioning and reduction.

**Figure 1:**
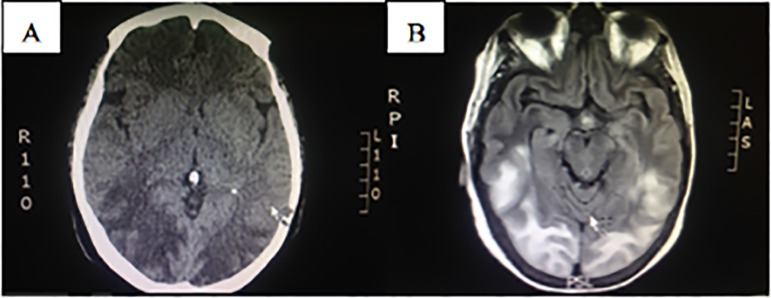
A. Brain CT scan disclosing extensive hypo-density at the sub cortical white and gray matter of the parietal and occipital lobes. Erasure of the cortical sulci, most evident on the cerebral hemispheres recesses. B. Magnetic resonance imaging disclosing hyperintensity on T2/FLAIR of the temporo-occipital and fronto-parietal regions in the upper convexity, without diffusion or bleeding signals.

Three days after discharge she was admitted to the emergency room complaining of severe headache, visual blurring, and confusion. Blood pressure was 180/100 mmHg, axillary temperature 38°C, and the general physical examination revealed no abnormalities. On neurological examination, she was confused, hallucinating, and disoriented. She presented transient visual alterations and left hemianopia without signs of meningeal irritation. Laboratory work up revealed stable graft function (serum creatinine 1.53 mg/dL), anemia (hemoglobin 7.7 g/dL) with normal white blood cell counts, slightly increased C reactive protein (10 mg/dL) and 10.3 ng/mL tacrolimus blood level. She received iv esmolol for blood pressure control and empiric iv antibiotics until cultures results. A brain CT scan disclosed extensive hypo-density at the sub cortical white and gray matter of the parietal and occipital lobes. Erasure of the cortical sulci, most evident on the cerebral hemispheres recesses, was also present, and PRES was considered in the differential diagnosis (Figure 1A). The magnetic resonance imaging (MRI) showed hyperintensity on T2/FLAIR of the temporo-occipital and fronto-parietal regions in the upper convexity, without diffusion or bleeding signals (Figure 1B).

Tacrolimus was discontinued from immunosuppressive therapy. In the next two days, the patient had complete reversal of neurological symptoms. Cyclosporine was started at 100 mg twice a day reaching a blood level of 146 ng/mL. The graft function remained stable and at two years after transplantation, the patient is enjoying good general condition and good graft function (serum creatinine 1.2 mg/dL), protein-creatinine ratio on random urine sample of 0.35 mg/mg, without new episodes of altered mental status or other neurologic signs.

## DISCUSSION

PRES is associated with a variety of conditions including hypertensive encephalopathy, eclampsia, porphyria, hypomagnesemia, sepsis, chronic kidney disease, and use of immunosuppressive drugs, especially calcineurin inhibitors (CNI).^1^^,^ 2 The syndrome has been reported in solid organ and bone marrow transplant recipients.^3^^,^ 4 ^,^ 5 ^,^ 6 ^,^ 7 Its incidence after solid organ transplantation has been estimated to be around 0.5%.^1^^,^ 6 ^,^ 8 Most of the PRES reports involved patients receiving immunosuppressive therapy with CNIs (cyclosporine or tacrolimus), although mTOR inhibitors (sirolimus or everolimus) have also been implicated.^9^^,^ 10 ^,^ 11 Tacrolimus was predominantly used in the cases described; no apparent relationship with blood levels has been reported and discontinuation of offending drugs usually led to clinical improvement.^7^^,^ 9 ^,^ 11

PRES diagnosis requires a high level of suspicion. In a clinical setting, patient complaints and careful physical and especially neurologic examination are crucial in the diagnostic work up. Imaging tests, usually CT and especially MRI, support the diagnosis as they reveal the presence of edema of the gray and white matter, mainly in the occipital and parietal lobes and to a lesser extent in the frontal and temporal lobes, pons, cerebellum, and other locations.^1^ In the post-transplant setting, the differential diagnosis includes infections or autoimmune encephalitis, vasculitis, and malignant diseases of the nervous system.^12^ “Top of basilar” embolism causing simultaneous bilateral posterior cerebral artery territory infarction is another important differential diagnosis and MRI establishes the diagnosis.^13^ MRI findings provide the dominant pathophysiological aspect of the syndrome, which is endothelial dysfunction leading to cerebral edema.^1^^,^ 14 ^,^ 16

In the case reported here, PRES seemed to be associated with tacrolimus use since a rapid clinical improvement occurred upon its discontinuation. Our patient was never exposed to toxic levels of tacrolimus, which prevented us from trying a lower dosage regimen of such drug. Instead, we opted for the use of cyclosporine starting with low doses and controlling blood levels up to the lowest therapeutic level. Our option for cyclosporine was based on the lower incidence of neurotoxicity in patients receiving cyclosporin as compared to tacrolimus.^7^^,^ 8 ^,^ 9 However we must recognize that recurrence could have occurred with cyclosporine therapy.

Two main approaches have been described for the handling of the syndrome in solid organ transplant recipients: discontinuation of the putative offending drug and dose reduction.^7^^,^ 8 ^,^ 9 In the first approach, the drug is usually replaced by another immunosuppressive agent, as was the case in our report; in the second possibility, the lowest effective drug level should be targeted.

PRES pathophysiology remains not fully elucidated. Two main hypotheses have been proposed both related to changes in cerebral blood flow.^8^^,^ 12 ^,^ 13 ^,^ 17 In short, it is currently accepted that hypertension and transient failure in self-regulation of cerebral blood flow causes vasogenic edema. Both neurogenic and myogenic responses lead to cerebral vessel vasodilation, and subsequent fluid leakage into the brain parenchyma.^1^^,^ 11 ^,^ 15 However, some patients have normal blood pressure, and an alternate explanation would be related to endothelial damage and dysfunction, followed by vasoconstriction leading to cerebral hypoperfusion.^6^^,^ 12 ^,^ 17 Likewise, the pathophysiology of PRES associated with immunosuppressive and cytotoxic drugs remains uncertain. It is thought to occur due to a direct toxic effect that damages the vascular endothelium, leading to endothelial dysfunction. This results in vasospasm, reduced tissue perfusion, activation of the coagulation cascade, and fluid leakage.^1^^,^ 12 ^,^ 17 It was initially suggested that an acute toxic insult of undetermined origin produced by these pharmacological agents results in axonal swelling and increased water content in the white matter.^6^^,^ 15 ^,^ 16 Alternatively, it has been proposed that vascular spasm, secondary to raised endothelin concentrations, might produce reversible ischemia. 12 ^,^ 17 ^,^ 18

Previous studies have indicated that polymorphisms in drug metabolizing genes could explain the propensity towards neurotoxicity. Tacrolimus is a substrate for the P-glycoprotein efflux pump encoded by the multidrug resistance gene-1. Although tacrolimus is lipophilic, it does not cross the blood-brain barrier by the action of the P-glycoprotein efflux pump. However, polymorphisms that impair the proper function of the efflux pumps may allow tacrolimus to enter the blood-brain barrier and therefore cause toxicity.^7^^,^ 19

Persistent neurological damage is reported in 10-20% of the patients and, even though PRES is not a frequent complication after transplantation, its early recognition and withdrawal of the offending immunosuppressive agent is crucial since delays in these measures may lead to significant morbidity and mortality while timely recognition and intervention usually leads to full recovery.^6^^,^ 7 ^,^ 8

In conclusion, our patient presented tacrolimus-related PRES, which was diagnosed early showing full recovery upon tacrolimus discontinuation. PRES should be considered in the differential diagnosis of solid organ transplant recipients with neurological symptoms in order to identify the syndrome and provide appropriate support and treatment.
